# Prenatal and Postnatal Bisphenol A Exposure and Body Mass Index in Childhood in the CHAMACOS Cohort

**DOI:** 10.1289/ehp.1205548

**Published:** 2013-02-15

**Authors:** Kim G. Harley, Raul Aguilar Schall, Jonathan Chevrier, Kristin Tyler, Helen Aguirre, Asa Bradman, Nina T. Holland, Robert H. Lustig, Antonia M. Calafat, Brenda Eskenazi

**Affiliations:** 1 Center for Environmental Research and Children’s Health, School of Public Health, University of California, Berkeley, Berkeley, California, USA; 2 Division of Endocrinology, University of California, San Francisco, San Francisco, California, USA; 3 Division of Laboratory Sciences, National Center for Environmental Health, Centers for Disease Control and Prevention, Atlanta, Georgia, USA

**Keywords:** bisphenol A, BMI, CHAMACOS, children, obesity

## Abstract

Background: Bisphenol A (BPA), a widely used endocrine-disrupting chemical, has been associated with increased body weight and fat deposition in rodents.

Objectives: We examined whether prenatal and postnatal urinary BPA concentrations were associated with body mass index (BMI), waist circumference, percent body fat, and obesity in 9-year-old children (*n* = 311) in the CHAMACOS longitudinal cohort study.

Methods: BPA was measured in spot urine samples collected from mothers twice during pregnancy and from children at 5 and 9 years of age.

Results: Prenatal urinary BPA concentrations were associated with decreased BMI at 9 years of age in girls but not boys. Among girls, being in the highest tertile of prenatal BPA concentrations was associated with decreased BMI *z*-score (β = –0.47, 95% CI: –0.87, –0.07) and percent body fat (β = –4.36, 95% CI: –8.37, –0.34) and decreased odds of overweight/obesity [odds ratio (OR) = 0.37, 95% CI: 0.16, 0.91] compared with girls in the lowest tertile. These findings were strongest in prepubertal girls. Urinary BPA concentrations at 5 years of age were not associated with any anthropometric parameters at 5 or 9 years, but BPA concentrations at 9 years were positively associated with BMI, waist circumference, fat mass, and overweight/obesity at 9 years in boys and girls.

Conclusions: Consistent with other cross-sectional studies, higher urinary BPA concentrations at 9 years of age were associated with increased adiposity at 9 years. However, increasing BPA concentrations in mothers during pregnancy were associated with decreased BMI, body fat, and overweight/obesity among their daughters at 9 years of age.

Bisphenol A (BPA) is a high-production chemical used in the manufacture of polycarbonate plastics, epoxy resins, and other industrial polymers. BPA can be present in a wide range of consumer products, including polycarbonate water bottles and food storage containers, epoxy-lined food cans, dental sealants, and thermal receipts. (Geens et al. 2011; Vandenberg et al. 2007). Exposure to BPA is almost ubiquitous, with > 90% of Americans having detectable urinary concentrations of BPA (Calafat et al. 2008). Although the main route of exposure to BPA is thought to be oral via diet, dermal and inhalation exposure are also possible (Biedermann et al. 2010; Stahlhut et al. 2009; Zalko et al. 2011).

BPA is an endocrine disruptor that may act as an estrogen at doses within the range of human exposure and may also interfere with androgens, thyroid hormones, and cell signaling pathways (Wetherill et al. 2007). Multiple studies in rodents have found that prenatal and early postnatal BPA exposure is associated with increased body weight and fat deposition (Akingbemi et al. 2004; Hiyama et al. 2011; Howdeshell et al. 1999; Miyawaki et al. 2007; Nikaido et al. 2004; Patisaul and Bateman 2008; Rubin et al. 2001; Somm et al. 2009; Wei et al. 2011; Xu et al. 2011). However, some studies find no associations (Newbold et al. 2007; Ryan and Vandenbergh 2006; Ryan et al. 2010) and others find decreased body weight (Alonso-Magdalena et al. 2010; Honma et al. 2002; Nagel et al. 1997; Nakamura et al. 2011; Negishi et al. 2003; Tyl et al. 2002). Although most studies have examined only perinatal exposure to BPA, Akingbemi et al. (2004) compared perinatal and chronic postnatal exposure with BPA in male rats and reported that perinatal exposure at 2.4 µg/kg/day was associated with increased body weight in adulthood but postnatal exposure (from weaning to adulthood) was not.

A small number of studies have examined the association of BPA and obesity in humans. Cross-sectional analyses of the National Health and Nutrition Examination Survey (NHANES) show that both children and adults with urinary BPA concentrations in the second, third and fourth quartiles had higher odds of obesity and larger waist circumference than those in the lowest quartile (Carwile and Michels 2011; Shankar et al. 2012; Trasande et al. 2012). Similarly, a cross-sectional study in China found that among adults, higher urinary BPA concentrations were positively associated with overweight, abdominal obesity, insulin resistance, and diabetes (Ning et al. 2011; Wang et al. 2012). However, all existing studies have been cross-sectional analyses. No studies have examined prenatal or early-life BPA exposure and subsequent adiposity in children. We examined the association of urinary BPA concentrations in pregnant women and their children in early childhood with body mass index (BMI), obesity, waist circumference, and percent body fat up to 9 years of age.

## Methods

*Study design and sample*. The Committee for the Protection of Human Subjects at the University of California, Berkeley, and at the Centers for Disease Control and Prevention (CDC) approved all study activities. The study sample consisted of participants in the Center for the Health Assessment of Mothers and Children of Salinas (CHAMACOS), a longitudinal cohort study of environmental factors and children’s growth and development. We enrolled pregnant mothers in 1999 and 2000 from prenatal clinics serving the farmworker population in the Salinas Valley, California. Eligible women were at least 18 years of age, spoke English or Spanish, qualified for low-income health insurance, were at < 20 weeks gestation, and were planning to deliver at the county hospital. Mothers provided written informed consent for themselves and their children to participate in the study.

Of 601 pregnant women enrolled in the study, a total of 527 were followed through the birth of a singleton, live-born infant. BPA measurements in spot urine collected during pregnancy were available for 498 mothers, and 402 children had at least one measure of BMI between 2 and 9 years of age.

*Data collection*. We interviewed mothers twice during pregnancy, after delivery, and when their children were 2, 3.5, 5, 7, and 9 years of age to obtain information about demographic characteristics, diet, and behaviors. All interviews were conducted in English or Spanish using structured questionnaires. At the baseline interview, we asked mothers about their race/ethnicity, education, income, marital status, and number of years they had lived in the United States, as well as information about soda consumption, smoking, and alcohol and drug use during pregnancy. We calculated prepregnancy BMI from self-reported prepregnancy weight and measured height. If self-reported prepregnancy weight was unavailable or invalid, we used measured weight at first prenatal visit (*n* = 23) if the first prenatal visit occurred at or before 13 weeks gestation or used regression models to impute prepregnancy weight based on weight at all prenatal visits if the first prenatal visit occurred after 13 weeks (*n* = 16).

We collected information about child behaviors from the mother during follow-up interviews, including asking about her child’s soda consumption, time spent watching television on weekdays and weekend days, and how often her child ate fast food (“from restaurants like McDonalds, Burger King, or KFC”) and sweet snacks (“like candy, cookies, cake, or other sweet snacks”).

When the child was 9 years of age, clinical Tanner staging was conducted by trained research staff to determine stages of pubertal development. Children were considered to have entered puberty if they were stage 2 or above for breast development for girls or stage 2 or above for pubic hair or genital development for boys.

*BPA measurements*. We collected spot urine samples from mothers at two time points during pregnancy: near the end of the first (mean ± SD, 13.8 ± 5.0 weeks gestation) and second (mean ± SD, 26.4 ± 2.4 weeks gestation) trimester of pregnancy and from the children when they were 5 (mean ± SD, 5.1 ± 0.2 years) and 9 (mean ± SD, 9.4 ± 0.4 years) years of age. Urine samples were collected in polypropylene urine cups, aliquotted into glass vials, and frozen at –80°C until shipment to the CDC for analysis. Analysis of field blanks showed no detectable contamination by BPA using this collection protocol.

We used solid-phase extraction coupled to high performance liquid chromatography–isotope dilution tandem mass spectrometry (Ye et al. 2005) to measure total urinary BPA concentration (conjugated plus unconjugated). The limit of detection (LOD) was 0.4 μg/L. Concentrations < LOD for which a signal was detected were reported as measured. Concentrations < LOD with no signal detected were randomly imputed based on a log-normal probability distribution using maximum likelihood estimation (Lubin et al. 2004).

Specific gravity was measured with a refractometer (National Instrument Company Inc., Baltimore, MD) for the maternal urine samples, but was unavailable for the children’s samples. Thus, maternal concentrations were normalized for urinary dilution using urine specific gravity (Mahalingaiah et al. 2008), and child BPA concentrations were normalized by dividing by urinary creatinine concentration.

For maternal BPA, two urinary measures were available for 307 women and were averaged to better approximate exposure over the course of pregnancy. In the 95 women for whom only one BPA measurement was available, the single measurement was used in lieu of the average. Child concentrations were used as separate variables representing exposure at 5 and 9 years of age.

*Anthropometric measurements*. At each assessment point (age 2, 3.5, 5, 7, and 9 years), children were weighed and measured without jackets or shoes by trained study staff. We measured weight using a digital scale (Tanita Mother-Baby Scale model 1582; Tanita Corp., Arlington Heights, IL)and rounded to the nearest 0.1 kg. We measured height using a stadiometer (Seca 222; Seca, Chino, CA) and rounded to the nearest 0.1 cm. Starting at 5 years of age, we measured waist circumference at each visit by placing a measuring tape around the abdomen at the level of the iliac crest, parallel to the floor. Height and waist circumference measurements were conducted in triplicate and averaged for analysis. When the children were 9 years of age, we measured fat percentage using “foot-to-foot” bio-impedence technology with a Tanita TBF-300A body composition analyzer (Tanita Corp.).

BMI was calculated as weight (kilograms) divided by height squared (square meters) and compared with the sex-specific BMI-for-age percentile data issued by CDC in 2000 (National Center for Health Statistics 2005). Children who were ≥ 85th but < 95th percentile for their age and sex were classified as overweight, children ≥ 95th percentile were classified as obese. Age- and sex-standardized BMI *z*-scores were also generated using the CDC norms (Grummer-Strawn et al. 2010).

*Statistical analysis*. All analyses were conducted using Stata 11 (StataCorp, College Station, TX). We used multivariable linear regression to examine the association of BPA concentrations with continuous outcomes (BMI *z*-score, waist circumference, and percent body fat) and multivariable logistic regression for categorical outcomes (e.g., overweight/obese).

We first examined the association of prenatal, age 5, and age 9 urinary BPA concentrations with outcomes at age 9 years. We also examined the association of prenatal BPA concentrations with repeated measures of child growth between age 2 and 9 years, using generalized estimating equations (GEE). GEE models included an interaction term for the BPA and age (continuous in months) variables to allow the association to differ by child age. Because the *p*-value on the age interaction term was < 0.1, we kept the interaction term in the model and estimated BPA effect coefficients for each age using the Stata “lincom” postestimation command. Because animal studies indicate that associations of BPA and BMI may differ by sex, and that differences in body weight may not become apparent until sexual maturity, we tested for effect modification by sex and by puberty status at age 9 using α = 0.1.

Urinary BPA concentrations were analyzed as continuous and categorical variables. Continuous BPA variables were log-transformed to reduce the influence of outliers. Because of the relatively narrow range of exposure, log base 2 was used. During pregnancy and at 5 years, categorical BPA concentrations were classified by tertiles because very few individuals had concentrations < LOD. At 9 years of age, categorical BPA concentrations were classified as < LOD, ≥ LOD but below the median, and above the median. Both visual inspection of the associations using lowess plots and regression models using categorical exposure variables gave no indication that associations were not linear.

We identified potential confounders *a priori* using directed acyclic graphs. Potential confounders included maternal prepregnancy BMI, age, education, years of residence in the United States, smoking during pregnancy, soda consumption during pregnancy, and family income. Time-varying covariates considered were child consumption of soda, fast food, and sweets, television watching, environmental tobacco smoke exposure, and time spent playing outdoors, assessed at multiple times during childhood. We included covariates in the final models if they were associated with both exposure and any of the growth outcomes at *p*-value < 0.2 or if removing them changed the coefficient for the main BPA exposure variable by > 10%. Maternal age and prepregnancy BMI were analyzed as continuous variables. Other variables were categorized as shown in Table 1.

**Table 1:** Geometric mean (GM) and SD (GSD) of urinary BPA concentrations (µg/L) by demographic characteristics of the study population, CHAMACOS study, Salinas, California, 2000–2010.

	n (%)	GM (GSD)		
		Pregnancya	5 years	9 years
Maternal characteristics (at time of pregnancy) (n = 402)
Race/ethnicity
Non-Latina, white	5 (1.2)	>1.7 (2.0)	>4.3 (1.9)	0.9 (1.3)
Latina	393 (97.8)	1.1 (2.3)	2.5 (2.9)	1.5 (2.7)
Other	4 (1.0)	2.4 (1.4)	3.9 (1.7)	2.2 (1.9)
Household incomeb
Below or equal to poverty	249 (61.9)	1.1 (2.3)	2.7 (3.1)	1.5 (2.6)
Above poverty	153 (38.1)	1.2 (2.2)	2.2 (2.6)	1.6 (2.9)
Education
≤ 6th grade	177 (44.0)	1.1 (2.3)	2.1 (2.7)	1.5 (2.8)
7–12th grade	144 (35.8)	1.1 (2.3)	2.8 (3.2)	1.7 (2.4)
≥ High school graduate	81 (20.2)	1.3 (1.9)	2.9 (2.8)	1.4 (3.0)
Years of residence in the USA
≤ 5	193 (48.0)	1.0 (2.2)	2.5 (3.1)	1.5 (2.8)
6–10	98 (24.4)	1.2 (2.3)	2.0 (2.4)	1.5 (2.6)
≥ 11	66 (16.4)	1.3 (2.2)	2.8 (2.6)	1.4 (2.8)
Entire life	45 (11.2)	1.6 (2.3)*	3.4 (3.8)	2.1 (2.3)
Soda consumption during pregnancy
< 1/day	327 (81.3)	1.1 (2.3)	2.5 (2.8)	1.5 (2.7)
1/day	56 (13.9)	1.2 (2.0)	2.4 (2.6)	1.6 (2.5)
1/day	56 (13.9)	1.2 (2.0)	2.4 (2.6)	1.6 (2.5)
≥ 2/day	19 (4.7)	1.6 (2.5)	2.7 (6.3)	2.3 (2.7)
Smoking during pregnancy
No	382 (95.0)	1.2 (2.3)	2.5 (3.0)	1.5 (2.7)
Yes	20 (5.0)	1.1 (2.2)	2.4 (2.0)	1.7 (3.4)
Prepregnancy BMI
Underweight	2 (0.5)	1.4 (7.4)	6.8 (—)c	1.0 (2.0)
Normal	144 (35.9)	1.1 (2.3)	2.4 (2.7)	1.4 (2.7)
Overweight	159 (39.7)	1.1 (2.0)	2.6 (3.5)	1.6 (2.8)
Obese	96 (23.9)	1.3 (2.6)	2.5 (2.4)	1.6 (2.6)
Child characteristics (at 5 years of age) (n = 319)
Soda consumption (non-diet)
< 1/week	122 (38.2)	1.1 (2.3)	2.8 (3.2)	1.6 (2.9)
1–6/week	170 (53.3)	1.1 (2.2)	2.4 (2.7)	1.5 (2.4)
1–6/week	170 (53.3)	1.1 (2.2)	2.4 (2.7)	1.5 (2.4)
≥ 1/day	27 (8.5)	1.4 (2.1)	2.1 (3.0)	1.7 (3.0)
Fast food consumption
< 1 time/week	116 (36.4)	1.1 (2.3)	2.4 (2.7)	1.5 (2.8)
1–2 times/week	195 (61.1)	1.2 (2.2)	2.6 (3.1)	1.6 (2.6)
≥ 3 times/week	8 (2.5)	1.5 (2.9)	1.5 (1.9)	1.3 (1.6)
Sweet snacks consumption
< 1/day	85 (26.7)	1.0 (2.3)	2.3 (3.0)	1.6 (3.1)
1–2/day	128 (40.1)	1.0 (2.1)	2.8 (3.0)	1.5 (2.7)
≥ 2/day	106 (33.2)	1.3 (2.3)	2.3 (2.8)	1.6 (2.3)
Average daily TV time
< 1 hr/day	41 (14.2)	1.1 (2.3)	2.9 (2.9)	1.6 (2.3)
1–2 hr/day	98 (34.0)	1.1 (2.3)	2.3 (2.7)	1.5 (2.8)
≥ 2 hr/day	149 (51.8)	1.2 (2.2)	2.4 (3.1)	1.6 (2.9)
Child BMI
Underweight (< 5th percentile)	0 (0.0)	—	—	—
Normal (5th–85th percentile)	147 (46.1)	1.2 (2.2)	2.3 (2.8)	1.3 (2.8)
Overweight (85th–95th percentile)	66 (20.7)	1.1 (2.1)	2.6 (3.3)	1.7 (2.3)
Obese (> 95th percentile)	106 (33.2)	1.0 (2.1)	2.7 (2.8)	1.9 (2.6)
Child characteristics (at 9 years of age) (n = 311)
Soda consumption (non-diet)
< 1/week	129 (41.5)	1.2 (2.4)	2.5 (2.9)	1.4 (2.8)
1–6/week	151 (48.6)	1.1 (2.2)	2.6 (3.0)	1.6 (2.6)
≥ 1/day	31 (10.0)	1.2 (2.5)	2.5 (2.7)	2.2 (2.4)*
Fast food consumptiond
< 1 time/week	115 (37.1)	1.2 (2.5)	2.3 (2.6)	1.5 (2.7)
1–2 times/week	183 (59.0)	1.1 (2.1)	2.8 (3.1)	1.5 (2.7)
≥ 3 times/week	12 (3.9)	1.6 (2.3)	1.4 (2.5)	2.3 (2.1)
Sweet snacks consumption
< 1/day	260 (83.6)	1.2 (2.3)	2.6 (2.9)	1.6 (2.7)
1–2/day	44 (14.2)	1.2 (1.9)	2.6 (2.8)	1.2 (2.6)
≥ 2/day	7 (2.3)	0.8 (2.9)	1.8 (2.8)	1.1 (1.7)
Average daily TV timed
< 1 hr/day	44 (14.2)	1.3 (2.6)	2.0 (2.3)	1.6 (2.5)
1–2 hr/day	102 (33.0)	1.1 (2.2)	2.9 (2.7)	1.5 (2.9)
≥ 2 hr/day	163 (52.7)	1.2 (2.2)	2.5 (3.1)	1.6 (2.6)
Onset of puberty: girlsd
No	86 (56.6)	1.3 (2.3)	2.2 (2.6)	1.2 (3.0)
Yes	66 (43.4)	1.0 (2.3)	2.2 (2.5)	1.6 (2.6)
Onset of puberty: boysd
No	114 (83.8)	1.1 (2.2)	3.2 (3.5)	1.9 (3.0)
Yes	22 (16.2)	1.1 (1.9)	2.5 (2.4)	1.6 (2.6)
Child BMI
Underweight (< 5th percentile)	0 (0.0)	—	—	—
Normal (5th–85th percentile)	134 (43.1)	1.2 (2.2)	2.4 (3.0)	1.3 (2.9)
Overweight (85th–95th percentile)	54 (17.4)	1.2 (2.3)	2.2 (2.5)	1.7 (2.4)
Obese (> 95th percentile)	123 (39.6)	1.1 (2.4)	2.8 (3.1)	1.8 (2.5)*
aAverage of two pregnancy measures. bHousehold income above or below poverty was determined by comparing total household income with the federal poverty threshold for a household of that size (U.S. Census Bureau 2000). cGSD could not be calculated; only one observation. dMissing data on fast food consumption (n = 1), TV time (n = 2), onset of puberty (n = 14 girls, n = 9 boys). *p < 0.05 based on analysis of variance (ANOVA).

In sensitivity analyses we *a*) analyzed BPA concentrations unadjusted for urinary dilution (specific gravity or creatinine); *b*) reanalyzed our models controlling separately for three important prenatal exposures in this population: organochlorine pesticides [using prenatal serum concentrations of dichlorodiphenyldichloroethylene (DDE)], organophosphate pesticides (using prenatal urinary metabolites of organophosphate pesticides), and brominated flame retardants [using prenatal serum concentrations of polybrominated diphenyl ethers (PBDEs)]; *c*) analyzed the two prenatal BPA measurements separately, as early pregnancy (< 20 weeks) or late pregnancy (≥ 20 weeks) measurements; and *d*) used inverse probability weights to account for potential bias due to loss to follow-up (Hernan et al. 2004).

## Results

Most mothers in the study population were Latina (98%), had lived in the United States for ≤ 10 years at the time of pregnancy (72%), had less than a high school education (80%), and were living at or below the poverty threshold (62%). Median age at the time of pregnancy was 26 years (range, 18–43). More than half of mothers were overweight or obese before pregnancy (64%) (Table 1). BPA concentrations during pregnancy were higher in mothers who had lived longer in the United States, and BPA concentrations at 9 years of age were higher in children who were obese and who drank more soda. More than half of the children in this cohort were overweight or obese (BMI > 85th percentile). At age 5 and 9 years, approximately 63% of children ate fast food at least once per week, and 8–10% drank soda every day (Table 1). Sweet snack consumption decreased between age 5 and 9 years but television watching increased, with more than half of children watching at least 2 hr of television per day at age 9 years. By age 9, 43% of the girls had begun puberty, compared with 16% of boys.

Of the 402 children included in the analysis, anthropometric measurements were available on 369 children at 2 years, 323 at 3.5 years, 319 at 5 years, 324 at 7 years, and 311 at 9 years of age. Children who were lost to follow-up by age 9 were more likely to have mothers who smoked (9.9% vs. 3.5%) or drank at least two sodas per day (7.7% vs. 2.3%) during pregnancy. No significant differences in birth weight or BMI at age 2 years were seen when we compared children lost to follow-up with those who participated at age 9.

The distribution of urinary BPA concentration in the pregnant mothers and their children at 5 and 9 years of age is shown in Table 2. The median BPA concentration in mothers (1.0–1.1 µg/L) was lower than in all women (2.4 µg/L) and Mexican-American women (2.8 µg/L) in NHANES in 2003–2004 (CDC 2005). Similarly, the median BPA concentrations in children at age 5 (2.3 µg/L) and 9 years (1.6 µg/L), although higher than their mothers’, were lower than among all children (3.7 µg/L) and Mexican-American children (2.9 µg/L) 6–11 years of age in NHANES (CDC 2005). Prenatal and childhood BPA concentrations were not correlated (*r* = 0.02, *p* = 0.73 for prenatal and age 5 years; *r* = 0.08, *p* = 0.22 for prenatal and age 9 years).

**Table 2:** BPA concentrations (μg/L) in urine of pregnant mothers and children, CHAMACOS study, Salinas Valley, California 2000–2010.

	n	Percent > LOD	Geometric mean	25th percentile	50th percentile	75th percentile	95th percentile
Maternal
Early pregnancy (≤ 20 weeks)	347	81.6	1.0	0.5	1.0	1.7	4.6
Late pregnancy (> 20 weeks)	383	81.7	1.0	0.5	1.1	1.9	4.4
Average	402	—	1.1	0.7	1.1	1.8	4.5
Child
5 years old	325	97.9	2.5	1.3	2.3	4.6	16.3
9 years old	304	89.8	1.5	0.9	1.6	2.8	7.4
LOD = 0.4 µg/L.							

**Figure 1:**
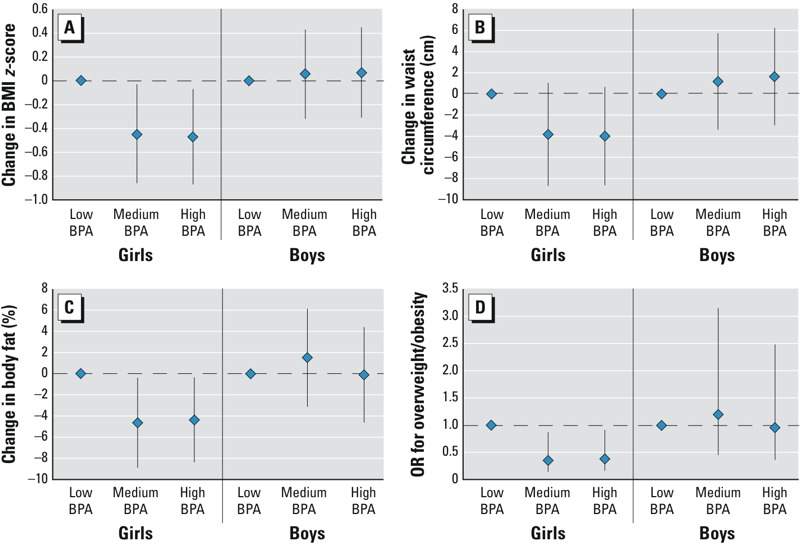
Sex-stratified associations of prenatal urinary BPA concentrations categories and changes in child body measurements at 9 years of age (*n* = 166 for girls; *n* = 145 for boys). (*A*) Prenatal BPA and BMI *z*-score by sex (sex × BPA interaction *p* = 0.05). (*B*) Prenatal BPA and waist circumference (sex × BPA interaction *p* = 0.08). (*C*) Prenatal BPA and body fat (sex × BPA interaction *p* = 0.11). (*D*) Prenatal BPA and obesity (sex × BPA interaction *p* = 0.15).

*Prenatal BPA and childhood body size*. The associations of urinary BPA concentrations measured during pregnancy with child body measurements at age 9 years are shown in Table 3. We found no significant associations between BPA concentrations during pregnancy with any measures of body size at age 9 looking at boys and girls combined, although the associations were consistently negative. However, associations appeared to be modified by sex, with negative associations in girls and no evidence of associations in boys (Figure 1). For example, the highest (vs. lowest) tertile of exposure was negatively associated in girls but not boys with BMI *z*-score (–0.47; 95% CI: –0.87, –0.07 and 0.07; 95% CI: –0.31, 0.45, respectively, interaction *p*-value = 0.05), waist circumference (–4.00 cm; 95% CI: –8.64, 0.65 and 1.64 cm; 95% CI: –2.96, 6.23; interaction *p*-value = 0.08), percent body fat (–4.36; 95% CI: –8.37, –0.34 and –0.10; 95% CI: –4.61, 4.40; interaction *p*-value = 0.11), and overweight/obesity [odds ratio (OR) = 0.38; 95% CI: 0.16, 0.91 and OR = 0.95; 95% CI: 0.36, 2.48; interaction *p*-value = 0.15]. Point estimates for all outcomes were similar for girls in the second and third tertiles of prenatal BPA concentration. We found no association of prenatal BPA with any body size parameters in boys.

**Table 3:** Association of BPA concentrations in maternal urine during pregnancy and child urine at 5 and 9 years of age with child’s body measurements at 9 years of age [β (95% CI)].

Concentration	n	BMI z-score	Waist circumference (cm)	Body fat (%)	Overweight/obese (> 85th percentile)
BPA during pregnancya,b
Log2 BPA (continuous)	311	–0.02 (–0.12, 0.08)	–0.12 (–1.29, 1.06)	–0.02 (–1.09, 1.04)	0.96 (0.76, 1.21)
Lowest tertile (< LOD–1.0 µg/L)	104	Reference	Reference	Reference	Reference
Middle tertile (1.0–1.7 µg/L)	102	–0.18 (–0.45, 0.09)	–1.20 (–4.37, 1.98)	–1.51 (–4.43, 1.41)	0.65 (0.35, 1.22)
Highest tertile (1.7–27.0 µg/L)	105	–0.23 (–0.50, 0.04)	–1.69 (–4.85, 1.47)	–2.35 (–5.20, 0.50)	0.56 (0.30, 1.04)
BPA at 5 yearsc,d
Log2 BPA (continuous)	274	0.01 (–0.08, 0.10)	–0.01 (–1.03, 1.06)	0.56 (–0.34, 1.46)	1.02 (0.84, 1.23)
Lowest tertile (< LOD–2.4 µg/g)	88	Reference	Reference	Reference	Reference
Middle tertile (2.4–4.5 µg/g)	97	0.05 (–0.26, 0.31)	0.27 (–3.14, 3.68)	0.86 (–2.20, 3.91)	0.91 (0.48, 1.73)
Highest tertile (4.6–349.8 µg/g)	89	0.14 (–0.14, 0.50)	0.93 (–2.56, 4.42)	1.82 (–1.37, 5.01)	1.28 (0.65, 2.51)
BPA at 9 yearsc,e
Log2 BPA (continuous)	290	0.04 (–0.07, 0.14)	0.87 (–0.35, 2.09)	0.44 (–0.66, 1.55)	1.06 (0.85, 1.33)
< LOD	30	Reference	Reference	Reference	Reference
Detectable < median (0.4–1.8 µg/g)	129	0.47 (0.07, 0.87)*	4.35 (–0.30, 9.02)*	3.56 (–0.74, 7.86)	3.08 (1.18, 8.02)*
Detectable > median (1.8–22.5 µg/g)	129	0.55 (0.15, 0.95)*	5.89 (1.19, 10.59)*	4.62 (0.26, 8.98)*	4.20 (1.60, 11.02)*
aAverage of two measures during pregnancy; adjusted for specific gravity to account for urinary dilution. bPrenatal models control for maternal prepregnancy BMI, household income, maternal education level, maternal years of residence in the United States, smoking during pregnancy, soda consumption during pregnancy, and child’s fast food and sweet consumption at age 9 years. cSingle measure at 5 and 9 years of age; adjusted for creatinine to account for urinary dilution. dFive-year models control for maternal prepregnancy BMI, household income, maternal education level, maternal years of residence in the United States, child’s environmental tobacco smoke exposure, soda intake, fast food intake, and sweet consumption at age 5 years. eNine-year models control for maternal prepregnancy BMI, household income, maternal education level, maternal years of residence in the United States, child’s environmental tobacco smoke exposure, soda intake, fast food intake, and sweet consumption at age 9 years. *p < 0.05.					

In exploratory analyses, we stratified by puberty status for girls but not boys, because too few boys had entered puberty by age 9 years. Despite small sample sizes, we observed interaction with puberty on the association of prenatal BPA concentrations and BMI *z*-score (interaction *p*-value = 0.04) in girls. Differences in effect by puberty status were also seen with waist circumference and percent body fat, although interaction terms were not statistically significant (interaction *p*-value = 0.11 for waist circumference and interaction *p*-value = 0.33 for percent body fat). In prepubertal girls, prenatal BPA was negatively associated with BMI *z*-score (β = –0.58; 95% CI: –1.15, –0.01), waist circumference (β = –6.16; 95% CI: –12.15, –0.18), and body fat (β = –4.61; 95% CI: –10.10, 0.88), comparing the highest versus lowest tertile of exposure, but not in girls who had begun puberty (β = 0.32; 95% CI: –0.45, 1.10 for BMI *z-*score; β = 2.33; 95% CI: –8.50, 13.16 for waist circumference; β = 1.12; 95% CI: –7.30, 9.55 for percent body fat) [see Supplemental Material, Figure S1 (http://dx.doi.org/10.1289/ehp.1205548)]. Similarly, when we estimated associations between prenatal BPA and the outcomes at 9 years of age after restricting the sample to children who had not yet begun puberty, the association of prenatal BPA concentrations with decreased BMI *z*-score, waist circumference, body fat, and obesity became slightly stronger both for all children and for girls in particular (see Supplemental Material, Table S1).

Longitudinal analyses of prenatal BPA concentration and childhood BMI *z*score between 2 and 9 years of age showed similar associations of decreased BMI *z*-score in girls but not boys [see Supplemental Material, Table S2 (http://dx.doi.org/10.1289/ehp.1205548)], although effect modification by sex was not statistically significant in this model (interaction *p*-value = 0.16). Similar findings were seen when the model was restricted to 2–7 years of age to remove the potential effect of children entering puberty by age 9 years (data not shown).

Prenatal BPA concentrations were not associated with birth weight or timing of puberty (data not shown).

*Postnatal BPA and childhood body size*. Urinary BPA concentrations at age 5 years were not associated with any body size parameters at age 9 (Table 3). However, BPA concentrations at age 9 were cross-sectionally associated with increased BMI *z*-score, waist circumference, and body fat and increased odds of obesity/overweight at age 9 after controlling for confounders. Compared with children who had no detectable concentrations of BPA, we observed increased odds of overweight/obesity (BMI *z*-score > 85th percentile) among children with 9-year BPA concentrations below the median (OR = 3.08; 95% CI: 1.18, 8.02) and among children with concentrations above the median (OR = 4.20; 95% CI: 1.60, 11.02) (Table 3). Interestingly, we observed no cross-sectional associations between BPA concentrations at age 5 and body size parameters at age 5 [see Supplemental Material, Table S3 (http://dx.doi.org/10.1289/ehp.1205548)]. We did not observe any effect modification by sex with childhood BPA measures and associations were similar for boys and girls.

*Sensitivity analyses*. We reran our models of prenatal, 5-year, and 9-year BPA concentrations with outcomes at age 9 and longitudinally between age 2 and 9 years without adjusting for urinary dilution. We found that the negative association of prenatal urinary BPA concentrations with 9-year body measures in girls was largely unchanged when we did not adjust for specific gravity and that the positive association of 9-year urinary BPA concentrations with BMI *z*-score, waist circumference, body fat, and obesity was considerably larger in magnitude when we did not adjust for creatinine (data not shown). When we controlled for prenatal concentrations of organophosphate pesticide metabolites (in urine) or DDE or PBDEs (in blood), results were virtually unchanged (data not shown). When we examined the two prenatal urine BPA measures separately, rather than the average of the two measures, we found no associations with the early pregnancy measure (≤ 20 weeks gestation). However, with the late pregnancy measure (> 20 weeks gestation), we found negative associations with BMI *z*-score that were similar to our findings with the average prenatal BPA concentration, suggesting that late pregnancy may be a more sensitive period of exposure (data not shown). Finally, adjusting for potential bias due to loss to follow-up using inverse probability weighting did not change the results substantially (data not shown).

## Discussion

In this low-income Mexican-American population, we found that higher BPA concentrations in children’s urine at 9 years of age were associated with increased odds of obesity and increased BMI *z*-score, waist circumference, and percent body fat at age 9. However, we found that higher prenatal urinary BPA concentrations were associated with decreased BMI *z*-scores, percent body fat, and obesity in girls at age 9 years. No association of prenatal BMI and body measurements was seen in boys. BPA concentrations in children’s urine at 5 years of age were not associated with any body size parameters at age 5 or 9 years.

Our finding of a cross-sectional association of BPA concentration and obesity at 9 years of age is consistent with cross-sectional studies of adults (Carwile and Michels 2011; Ning et al. 2011; Shankar et al. 2012; Wang et al. 2012) and older children (Trasande et al. 2012). In the only other study of BPA and obesity in children, Trasande et al. (2012) found that among 6- to 19-year-olds participating in NHANES, increasing urinary BPA concentrations were cross-sectionally associated with increased BMI *z*-score and increased odds of obesity. Although some of the children in that study were younger than our participants, most were older and most would have already entered puberty. The fact that we found similar results in 9-year-olds but not 5-year-olds suggests that this association may hold for adults and older children, but not for younger children.

As with the previous studies, the cross-sectional nature of our findings at age 9 years limits our ability to establish temporality. BPA concentrations may be a surrogate for other factors or characteristics (e.g., lifestyle, diet) that are themselves predictive of obesity. For example, overweight individuals may consume more canned soda or heavily packaged, processed foods, which are important sources of BPA. Thus, higher BPA may not be a cause of obesity but rather a by-product of dietary habits associated with obesity. Alternatively, because the main source of BPA is thought to be diet, it is possible that overweight individuals simply consume more calories than people with normal weight, resulting in higher urinary BPA concentrations than normal-weight individuals. It is also possible that overweight people metabolize BPA differently than those who are normal weight, resulting in the observed differences in urinary BPA concentrations. In our study population, small increases in BPA concentrations [i.e., comparing the medium group (0.4–1.8 ng/mL) to those < LOD (< 0.4 ng/mL)] at age 9 years were associated with very large increases in waist circumference (4.35 cm) and body fat (3.56%). This seems unlikely and may reflect reverse causality.

This is the first study to examine prenatal BPA exposure and childhood BMI. Our finding that mothers’ prenatal BPA concentrations are associated with decreased BMI *z*-score, waist circumference, body fat, and obesity in their daughters was unexpected and highlights the limitations of cross-sectional analyses. Although many animal studies have shown perinatal BPA exposure to be associated with increased body weight in mice and rat offspring (Akingbemi et al. 2004; Hiyama et al. 2011; Howdeshell et al. 1999; Miyawaki et al. 2007; Nikaido et al. 2004; Patisaul and Bateman 2008; Rubin et al. 2001; Somm et al. 2009; Wei et al. 2011; Xu et al. 2011), other animal studies show associations of BPA with decreased body weight (Alonso-Magdalena et al. 2010; Honma et al. 2002; Nagel et al. 1997; Nakamura et al. 2011; Negishi et al. 2003; Tyl et al. 2002) or no difference in weight (Newbold et al. 2007; Ryan and Vandenbergh 2006; Ryan et al. 2010). Results vary considerably depending on dose, sex, rodent model, timing of exposure, and timing of outcome, and comparisons across studies are difficult. Although decreased body weight in rat offspring has been observed at very high doses (> 4,000 µg/kg/day) (Negishi et al. 2003; Tyl et al. 2002), it has also been observed in animal studies using lower, more environmentally relevant doses of BPA (Alonso-Magdalena et al. 2010; Honma et al. 2002; Nagel et al. 1997; Nakamura et al. 2011).

Of the rodent studies that have found perinatal BPA exposure linked to postnatal weight gain and adiposity, many have found that the increase is found only at low doses and is not apparent until the animals reach sexual maturity (Akingbemi et al. 2004; Howdeshell et al. 1999; Miyawaki et al. 2007; Patisaul and Bateman 2008; Rubin et al. 2001; Wei et al. 2011). For example, Wei et al. (2011) dosed pregnant rats with 50, 250, or 1,250 µg/kg of BPA each day between gestational day 0 and postnatal day 21 and found no differences in body weight in early life; however, they found significantly increased body weight in the low-dose group compared with controls beginning around 8 weeks of life, as the animals approached adulthood. Thus, it is possible that the association of BPA exposure and BMI will change as the children in our study enter adolescence. The association of prenatal BPA with decreased BMI *z*-score in this study was strongest among girls who had not yet reached puberty. Additional analyses of these children and other children at older ages will help clarify the role of puberty in the observed associations.

The median urinary BPA concentrations in this study are approximately half those observed in the NHANES general population (Calafat et al. 2008; Woodruff et al. 2011). It is likely that the low BPA concentrations in this study hampered our ability to detect associations, and that stronger associations might be seen in populations with higher BPA concentrations. Additionally, because this Mexican-American cohort was recruited from clinics serving the farmworker population, other pollutants, including pesticides, may be confounders in the relationship between urinary BPA and BMI. However, controlling for DDE, organophosphate pesticides, and PBDEs did not change our results.

An additional limitation is the transient nature of BPA exposure assessment. Urinary BPA concentrations vary widely throughout the day (Ye et al. 2011), and any spot urine sample likely reflects only exposure in the previous 4–6 hr. Multiple urinary BPA measurements over time tend to exhibit larger within-person than between-person variability (Braun et al. 2011, 2012; Teitelbaum et al. 2008; Ye et al. 2011), a finding also observed in this sample (Quiros L, personal communication). Thus, it is very difficult to estimate long-term exposure from one spot urinary measurement. We averaged two measurements of maternal urinary BPA concentrations, one in early and one in late pregnancy, to better estimate exposure throughout the pregnancy, but even two measurements may be insufficient to accurately characterize on-going exposure.

We adjusted our maternal prenatal BPA concentrations by specific gravity to account for variability in urinary dilution. Unfortunately, specific gravity was not measured on the child urine samples, so we adjusted our child BPA concentrations by urinary creatinine instead. Creatinine excretion is dependent on individual physiological factors including muscle mass and growth, and may not be the best method of accounting for urinary dilution in children (Boeniger et al. 1993). Our cross-sectional finding that BPA at 9 years was associated with increased BMI and adiposity was stronger when we did not adjust for creatinine. In our sample, urinary creatinine concentrations were positively associated with BMI, which suggests that adjusting for creatinine in this case may not be appropriate because we may be controlling for a factor (creatinine) that is a direct consequence of the outcome of interest (BMI).

Despite the potential for misclassification of BPA exposure and the considerable “noise” in this measurement, we found associations of urinary BPA concentrations during pregnancy and at age 9 years with measures of BMI and adiposity. We found that prenatal BPA concentrations were negatively associated with BMI in girls between 2 and 9 years of age. This finding is contrary to cross-sectional studies that suggest that BPA is associated with increased obesity in children and adults. It is possible that cross-sectional analyses partly reflect reverse causality and that early-life BPA exposure is actually associated with decreased obesity in children. It is also possible that the obesogenic effects of BPA do not manifest until after puberty. Additional prospective studies of early-life BPA exposure that follow children into adolescence are needed.

## Supplemental Material

(504 KB) PDF
