# Supplementary material for: Prenatal and Postnatal Bisphenol A Exposure and Body Mass Index in Childhood in the CHAMACOS Cohort
Source: Environ Health Perspect. 2013 Feb 15;121(4):514–20. doi: 10.1289/ehp.1205548 (PMC3620748; doi:10.1289/ehp.1205548)
Supplement: (504 KB) PDF [file ehp.1205548.s001.pdf]

SUPPLEMENTAL MATERIAL

Prenatal and Postnatal Bisphenol A Exposure and Body Mass Index in Childhood in the CHAMACOS Cohort

Kim G. Harley, Raul Aguilar Schall, Jonathan Chevrier, Kristin Tyler, Helen Aguirre, Asa Bradman, Nina T. Holland, Robert H. Lustig, Antonia M. Calafat, and Brenda Eskenazi

Table of Contents

Supplemental Materials, Figure S1: Associations of prenatal urinary BPA concentration categories and changes in BMI Z-score at age 9 years in girls, stratified by puberty status. (N=86 prepubescent and N=66 for pubescent girls) 2

Figure S1A: Prenatal BPA and BMI Z-score at age 9 by puberty status 2

Figure S1B: Prenatal BPA and waist circumference at age 9 by puberty status 3

Figure S1C: Prenatal BPA and percent body fat at age 9 by puberty status 3

Supplemental Materials, Table S1: Association of BPA concentrations in maternal urine during pregnancy with child's body measurements at 9 years of age for prepubescent children only 4

Supplemental Materials, Table S2: Longitudinal association of average BPA concentrations in maternal urine during pregnancy and child's BMI Z-score at various ages 6

Supplemental Materials, Table S3: Association of BPA concentrations in child urine at age 5 with child's body measurements at 5 years of age. 7

Supplemental Materials, Figure S1: Associations of prenatal urinary BPA concentration categories and changes in BMI Z-score at age 9 years in girls, stratified by puberty status. (N=86 prepubescent and N=66 for pubescent girls)

Figure S1A: Prenatal BPA and BMI Z-score at age 9 by puberty status, (puberty x BPA interaction p=0.04)

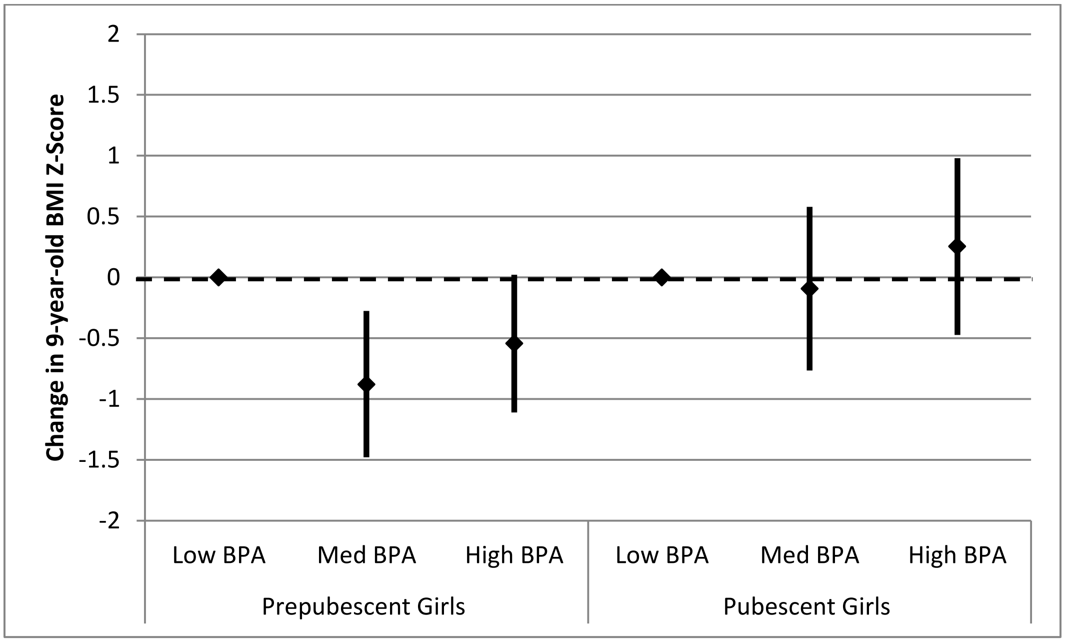

Figure S1B: Prenatal BPA and waist circumference at age 9 by puberty status, (puberty x BPA interaction p=0.11)

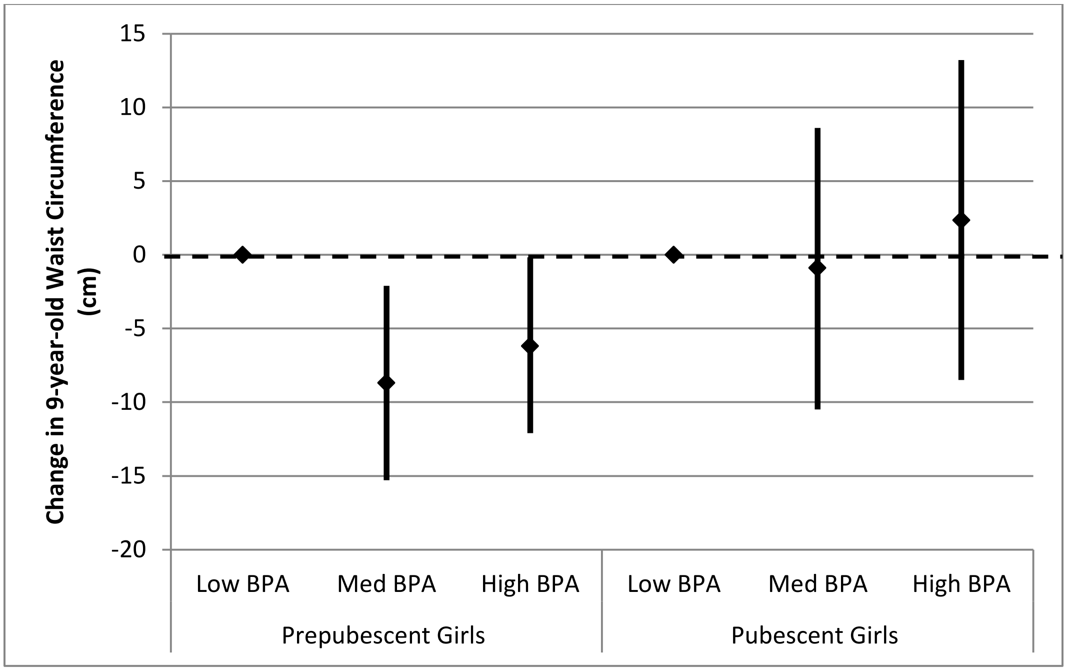

Figure S1C: Prenatal BPA and percent body fat at age 9 by puberty status, (puberty x BPA interaction p=0.33)

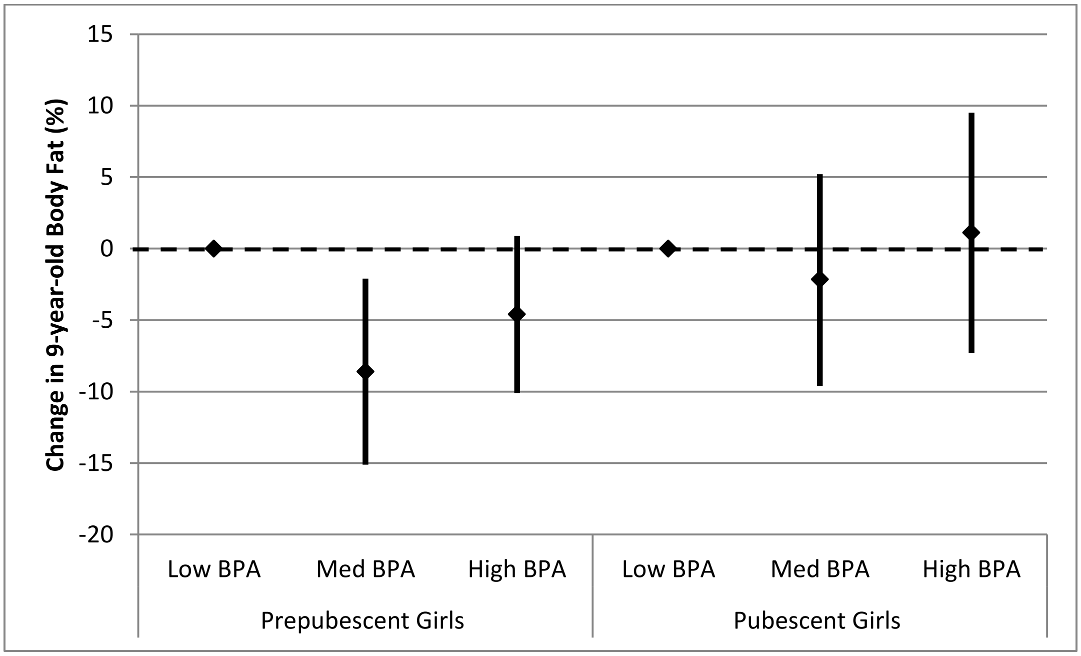

Supplemental Materials, Table S1: Association of BPA concentrations in maternal urine during pregnancy with child's body measurements at 9 years of age for prepubescent children only

	N	BMI Z-score β (95% CI)	Waist Circumference (cm) β (95% CI)	Body Fat (%) β (95% CI)	Overweight/Obese β (95% CI)
Prenatal BPA[a, b]					
Log_2 BPA (continuous)	200	-0.10 (-0.23, 0.03)	-0.76 (-2.19, 0.68)	-0.40 (-1.76, 0.95)	0.78 (0.58, 1.04) †
Lowest Tertile	68	Reference	Reference	Reference	Reference
Middle Tertile	59	-0.38 (-0.75, -0.02) *	-3.39 (-7.40, 0.62) †	-2.02 (-5.91, 1.87)	0.42 (0.19, 0.95) *
Highest Tertile	73	-0.34 (-0.69, 0.00) †	-2.65 (-6.48, 1.18)	-2.45 (-6.03, 1.13)	0.36 (0.16, 0.81) *
Prenatal BPA- GIRLS ONLY [a, b]					
Log_2 BPA (continuous)	86	-0.19 (-0.40, 0.03) †	-2.11 (-4.37, 0.15) †	-1.13 (-3.26, 1.00)	0.52 (0.30, 0.89) *
Lowest Tertile	25	Reference	Reference	Reference	Reference
Middle Tertile	25	-0.92 (-1.54, -0.29) *	-8.68 (-15.29, -2.06) *	-8.62 (-15.11, -2.13) *	0.15 (0.03, 0.73) *
Highest Tertile	36	-0.58 (-1.15, -0.01) *	-6.16 (-12.15, -0.18) *	-4.61 (-10.10, 0.88) †	0.14 (0.03, 0.65) *
Prenatal BPA- BOYS ONLY [a, b]					
Log_2 BPA (continuous)	114	0.07 (-0.10, 0.24)	0.90 (-1.17, 2.97)	0.46 (-1.49, 2.42)	1.20 (0.77, 1.87)
Lowest Tertile	43	Reference	Reference	Reference	Reference
Middle Tertile	34	0.01 (-0.44, 0.47)	0.67 (-4.86, 6.20)	2.30 (-3.11, 7.72)	0.96 (0.32, 2.86)
Highest Tertile	37	0.50 (-0.41, 0.51)	1.52 (-4.09, 7.12)	-0.54 (-5.74, 4.65)	0.99 (0.32, 3.08)

[a] Average of two measures during pregnancy. Adjusted for specific gravity to account for urinary dilution.

[b] All models control for maternal pre-pregnancy BMI, household income, maternal education level, maternal years of residence in the US, smoking during pregnancy, soda consumption during pregnancy, and child's fast food and sweet consumption at age 9.

* p-value < 0.05 † p-value <0.1

Supplemental Materials, Table S2: Longitudinal association of average BPA concentrations in maternal urine during pregnancy and child's BMI Z-score at various ages

			BMI Z-Score				
			2 Years	3.5 Years	5 Years	7 Years	9 Years
	N_{obs}	N_{ind}	β (95% CI)	β (95% CI)	β (95% CI)	β (95% CI)	β (95% CI)
Prenatal BPA-BOYS ONLY [a, b]							
Log_2 BPA (continuous)	772	192	-0.07 (-0.22, 0.09)	-0.04 (-0.18, 0.10)	-0.01 (-0.13, 0.12)	0.03 (-0.09, 0.16)	0.07 (-0.07, 0.22)
Lowest Tertile	556	131	Reference	Reference	Reference	Reference	Reference
Middle Tertile	524	132	-0.18 (-0.59, 0.23)	-0.12 (-0.49, 0.25)	-0.06 (-0.40, 0.28)	-0.02 (-0.31, 0.35)	0.11 (-0.25, 0.46)
Highest Tertile	537	133	-0.16 (-0.58, 0.25)	-0.12 (-0.49, 0.25)	-0.08 (-0.42, 0.26)	-0.03 (-0.37, 0.31)	0.03 (-0.34, 0.40)
Prenatal BPA-GIRLS ONLY [a, b]							
Log_2 BPA (continuous)	1,617	395	-0.16 (-0.28, -0.05)*	-0.15 (-0.25, -0.04)*	-0.13 (-0.23, -0.02)*	-0.10 (-0.22, 0.01)†	-0.08 (-0.23, 0.07)
Lowest Tertile	556	131	Reference	Reference	Reference	Reference	Reference
Middle Tertile	524	132	-0.25 (-0.63, 0.14)	-0.30 (-0.65, 0.05)	-0.36 (-0.68, -0.03)*	-0.43 (-0.76, -0.10)*	-0.50 (-0.87, 0.14)*
Highest Tertile	537	133	-0.33 (-0.68, 0.02)†	-0.33 (-0.67, 0.01) †	-0.34 (-0.65, -0.03)*	-0.35 (-0.67, -0.03)*	-0.36 (-0.71, 0.01) †

[a] Average of two measures during pregnancy. Adjusted for specific gravity to account for urinary dilution.

[b] All models control for child's age in exact months, maternal pre-pregnancy BMI, household income, maternal education level, maternal years of residence in the US, smoking during pregnancy, soda consumption during pregnancy, and child's fast food consumption at time of growth assessment. Analyses used generalized estimating equations (GEE) with interaction term for BPA*age

Abbreviations: N_{obs}: Number of observations, N_{ind}: Number of individuals * p-value < 0.05 † p-value <0.1

Supplemental Materials, Table S3: Association of BPA concentrations in child urine at age 5 with child's body measurements at 5 years of age.

	N	BMI Z-score β (95% CI)	Waist Circumference (cm) β (95% CI)	Overweight/Obese (>85th %ile) OR (95% CI)
BPA at 5 Years [d, e]				
Log_2 BPA (continuous)	310	0.02 (-0.07, 0.11)	0.13 (-0.50, 0.76)	1.07 (0.90, 1.28)
Lowest Tertile	105	Reference	Reference	Reference
Middle Tertile	104	-0.06 (-0.35, 0.24)	-0.08 (-2.14, 1.98)	0.80 (0.45, 1.42)
Highest Tertile	102	0.16 (-0.14, 0.46)	1.08 (-1.02, 3.18)	1.36 (0.75, 2.47)

[d] Adjusted for creatinine to account for urinary dilution

[e] All models control for maternal pre-pregnancy BMI, household income, maternal education level, maternal years of residence in the US, child's tobacco smoke exposure, soda intake, fast food intake, and sweet consumption at age 5.

* p-value < 0.05 † p-value <0.1
